# Prognostic role of cyclin D2/D3 in multiple human malignant neoplasms: A systematic review and meta‐analysis

**DOI:** 10.1002/cam4.2152

**Published:** 2019-04-05

**Authors:** Zuo‐you Ding, Ran Li, Qi‐jie Zhang, Yi Wang, Yi Jiang, Qing‐yang Meng, Qiu‐lei Xi, Guo‐hao Wu

**Affiliations:** ^1^ Department of General Surgery Zhongshan Hospital, Fudan University Shanghai China; ^2^ Department of Urology The First Affiliated Hospital of Nanjing Medical University Nanjing China

**Keywords:** Cyclin D2, Cyclin D3, meta‐analysis, prognosis

## Abstract

Cyclin D2/D3 (CCND2/3) are core components of the machinery that drives cell cycle progression and therefore, are associated with tumorigenesis. Currently, there are contradictory evidences on the function of CCND2/3 in tumorigenesis. Thus, we conducted a comprehensive meta‐analysis to derive a precise predictive value of CCND2/3 in various tumors. We searched PubMed, EMBASE, Web of Science for eligible studies up to October 8, 2018. Pooled hazard ratios (HRs) with 95% confidence intervals (CIs) of OS or DFS/PFS/RFS were calculated using Forest plot analysis to demonstrate their associations. A total of 14 studies were ultimately included in this meta‐analysis. Our results indicated CCND2/3 played an oncogenic role in all of the cancer patients (CCND2: pooled HR = 2.21, 95% CI: 1.67‐2.93; CCND3: pooled HR = 2.29, 95% CI: 1.05‐5.03). In tumor subgroup, CCND2 was associated with shorter OS in patients with gastric cancer (HR = 2.20, 95% CI: 1.66‐2.92), whereas it might be a tumor suppressor in NSCLC (HR = 0.28, 95% CI: 0.12‐0.64). In addition, CCND3 was correlated to reduced OS in breast cancer patients (HR = 1.64, 95% CI: 1.07‐2.52) and shorter DFS/PFS/RFS in bladder cancer patients (HR = 4.60, 95% CI: 1.89‐12.57). Taken together, CCND2/3 could be the promising biomarkers for predicting the prognosis of patients with malignant neoplasms.

## 1 INTRODUCTION

D‐type cyclins (D1, D2, and D3), as the regulatory partners for cyclin‐dependent kinases 4 and 6 (CDK4 and CDK6), are essential in cell cycle progression. Cyclin–CDK complexes promote a plethora of cellular proteins entry into G1 phase. It also coordinates the sequential completion of DNA replication and in turn determines cell division, thereby ensuring that the cell cycle progresses in an ordered manner.^1^, ^2^ Abnormal expression of cyclins has been reported in nearly all human tumor types.^3^, ^4^

Cyclin D1 (CCND1) is an established oncogene. The involvement of CCND1 amplification and overexpression is associated with tumor differentiation, increased metastasis, and poor survival.^5^ Cyclin D2 (CCND2) overexpression has been noted in gastric cancer.^6^ Targeting Cyclin D2 by miR‐4317 can reduce non‐small cell lung cancer (NSCLC) cell growth and metastasis.^7^ In addition, leukemic fusion protein RUNX1/ETO drives leukemic transformation via regulating CCND2 expression.^8^ Cyclin D3 (CCND3) plays an important role in the cell cycle by controlling physiological progression from G1 to S phase and the deregulation of CCND3 has been confirmed to be associated with the development of some malignant tumors.^9^ miR‐195 targets cyclin D3 to cause cell cycle arrest at the G1 phase, resulting in the reduction of NSCLC cell growth.^10^ Furthermore, Chi et al reported that cyclin D3 could serve as an independent prognosis marker in breast cancer.^11^

Several meta‐analysis articles have explored the prognostic role of CCND1 in breast cancer,^12^ gastric cancer,^13^ bladder cancer,^14^ lung cancer,^15^ and oral squamous cell carcinomas.^16^ However, no meta‐analysis for CCND2 or CCND3 has been conducted. In previous studies, CCND2 and CCND3 have been identified to be involved in oncogenesis, whereas the role of them were inconsistent and inconclusive in several studies.^6^, ^17^, ^18^ Hence, we conducted a meta‐analysis including all eligible case‐control studies to investigate the prognostic role of cyclin D2/D3 in multiple human malignant neoplasms.

## 2 METHODS

### 2.1 Data from GEPIA, KM‐plotter, and the human protein atlas database acquisition

The prognostic data of CCND2/3 were from KM‐plotter (http://kmplot.com/analysis/).^19^ We downloaded CCND2/3 expression data on patients with various cancers from Gene Expression Profiling Interactive Analysis (http://gepia.cancer-pku.cn/).^20^ In addition, the translational‐level CCND2/3 was downloaded from The Human Protein Atlas Database (https://www.proteinatlas.org/).^21^

### 2.2 Search strategy

We searched PubMed, EMBASE, Web of Science to identified relevant literature published up to October 8, 2018. The searching key words for this literature retrieval were as follows: (“cancer” or “carcinoma” or “neoplasm” or “tumor” or “tumour”) and (“Cyclin D2” or “Cyclin D3”) and (“prognostic” or “prognosis” or “survival” or “outcome” or “recurrence” or “relapse”).

### 2.3 Inclusive and exclusive criteria

All eligible articles were selected according to the following inclusion criteria: (a) Human subjects, English publications; (b) Independent case‐control or cohort studies; (c) Possessing at least one of D‐type cyclins (D2 or D3); (d) Availability of hazard ratios (HRs) for prognostic outcomes data of both cases and controls. The exclusion criteria were as follows: (a) No case‐control study; (b) Duplicate or unavailable data; (c) Studies not related to Cyclin D2 or Cyclin D3.

### 2.4 Quality assessment

Two blind investigators assessed the quality of all included studies according to the Newcastle‐Ottawa Scale (NOS) system, which is one of the most useful scale to evaluate the quality of non‐randomized studies in meta‐analysis.^22^ The criteria of quality assessment are as follows: (a) representativeness of the exposed cohort; (b) selection of the non‐exposed cohort; (c) ascertainment of exposure; (d) outcome of interest not present at start of study; (e) control for important factor or additional factor; (f) assessment of outcome; (g) follow‐up long enough for outcomes to occur; (h) adequacy of follow‐up of cohorts. Studies with a total score of ≤5 stars, 6‐7 stars, and 8‐9 stars were considered to be of low quality, intermediate quality, and high quality, respectively. All included studies had an intermediate or high quality according to NOS.

### 2.5 Data extraction

All data from eligible studies were extracted independently. Ambiguous data were reviewed in detail between authors to reach a consensus. All the extracted data were recorded in a unified format and the collected items were as follows: first author' name, publication year, patients' median or mean age, nationality, dominant ethnicity, the number of patients, investigating method, follow‐up time, cutoff value, and hazard ratios (HRs) for prognostic outcomes (overall survival [OS] and disease/recurrence/progression free survival [DFS/RFS/PFS]) along with their 95% CI and *P*‐values. Data were extracted from Kaplan–Meier curves to extrapolate HRs with 95% CIs using previously described methods, if it could not be directly obtained from each article.^23^, ^24^ All of the aforementioned data are comprehensively detailed in Tables 1 and 2.

**Table 1:** Main characteristics of studies included in the meta‐analysis

First author, publication year	Case nationality	Dominant ethnicity	Median or mean age	Study design	Malignant disease	Main type of pathology	Detected sample	Assay method	Survival analysis	Source of HR	Maximum months of follow‐up	NOS scores
CCND2												
Shan, 2017	China	Asian	NM	R	Gastric cancer	Adenoca	Tissue	IHC	OS^U^/PFS^U^	Reported	NM	7
Ko, 2012	Korea	Asian	60.5	R	NSCLC	Sqca	Tissue	IHC	RFS^U^	Reported	64	8
Mitra, 2011	America	Caucasian	61.8	R	NSCLC	Adenoca	Tissue	IHC	RFS^U^	Reported	67	7
Sarkar, 2009	Britain	Caucasian	65.1	R	Colorectal cancer	Adenoca	Tissue	IHC	OS^U^/DFS^U^	SC	89	7
Takano, 2000	Japan	Asian	60.9	R	Gastric cancer	Adenoca	Tissue	IHC	OS^U^	SC	65	6
Takano, 1999	Japan	Asian	60.6	R	Gastric cancer	Adenoca	Tissue	IHC	OS^U^	SC	63	6
CCND3												
Shan, 2017	China	Asian	NM	R	Gastric cancer	Adenoca	Tissue	IHC	OS^U^/PFS^U^	Reported	NM	7
Chi, 2015	China	Asian	NM	R	Breast cancer	Adenoca	Tissue	IHC	OS^U^/DFS^U^	SC	88	8
Sarkar, 2009	Britain	Caucasian	65.1	R	Colorectal cancer	Adenoca	Tissue	IHC	DFS^U^	SC	85	7
Sterlacci, 2010	Austria	Caucasian	62	R	NSCLC	Adenoca	Tissue	IHC	OS^U^	SC	168	6
Levidou, 2007	Greece	Caucasian	57	R	Ovarian cancer	Serous	Tissue	IHC	OS^M^	Reported	126	7
Filipits, 2007	Mixed	Mixed	NM	R	NSCLC	Adenoca	Tissue	IHC	OS^M^	Reported	NM	7
Lopez‐Beltran, 2006	Spain	Caucasian	61	R	Bladder cancer	Sqca	Tissue	IHC	PFS^U^	SC	120	6
Galizia, 2006	NM	Mixed	NM	R	Gastric cancer	Adenoca	Tissue	IHC	DFS^U^	Reported	NM	7
Lopez‐Beltran, 2004	Spain	Caucasian	70	R	Bladder cancer	Sqca	Tissue	IHC	OS^U^/DFS^U^/PFS^U^	SC	144	6
Keyomarsi, 2002	America	Caucasian	64	R	Breast cancer	Adenoca	Tissue	IHC	OS^U^	SC	132	6

**Table 2:** HRs and 95% CIs of patient survival or cancer progression relating to CCND2/3 expression in eligible studies

First author, publication year	Assay method	Cutoff point	Case number		OS		DFS/RFS/PFS	
			High expression	Low expression	HR (95% CI)	*P*‐value	HR (95% CI)	*P*‐value
CCND2								
Shan, 2017	IHC	NM	NM	NM	0.67 (0.55‐0.82)	0.00011	0.64 (0.51‐0.82)	0.0003
Ko, 2012	IHC	>10% of tumor cells stained	91	35	NM	NM	0.27 (0.08‐0.65)	0.03
Mitra, 2011	IHC	>10% of tumor cells stained	NM	NM	NM	NM	0.30 (0.08‐1.16)	NM
Sarkar, 2009	IHC	>5% of tumor cells stained	24	22	2.72 (1.23‐31.60)	0.002	5.23 (2.16‐21.90)	0.022
Takano, 2000	IHC	>15% of tumor cells stained	43	73	1.96 (1.16‐2.93)	0.03	NM	NM
Takano, 1999	IHC	>15% of tumor cells stained	105	350	2.36 (1.65‐3.39)	<0.0001	NM	NM
CCND3								
Shan, 2017	IHC	NM	NM	NM	0.61 (0.31‐1.2)	0.14	0.46 (0.17‐1.25)	0.12
Chi, 2015	IHC	>5% of tumor cells stained	170	73	1.43 (0.13‐16.67)	0.088	4.71 (1.81‐9.06)	0.001
Sterlacci, 2010	IHC	scores > 3.5 (range of 0‐90)	167	224	1.45 (1.17‐1.61)	0.019	NM	NM
Sarkar, 2009	IHC	>5% of tumor cells stained	20	26	NM	NM	3.42 (1.56‐10.46)	0.016
Levidou, 2007	IHC	>5% of tumor cells stained	NM	NM	0.16 (0.04‐0.62)	0.008	NM	NM
Filipits, 2007	IHC	NM	358	181	1.04 (0.84‐1.28)	0.74	NM	NM
Lopez‐Beltran, 2006	IHC	>25% of tumor cells stained	9	150	NM	NM	5.02 (3.01‐76.66)	<0.0001
Galizia, 2006	IHC	scores > 3 (range of 0‐12)	NM	NM	NM	NM	1.297 (0.50‐3.36)	0.592
Lopez‐Beltran, 2004	IHC	>15% of tumor cells stained	5	46	6.96 (2.79‐11.09)	<0.0001	4.36 (1.81‐23.47)	0.0168
Keyomarsi, 2002	IHC	>10% of tumor cells stained	115	63	1.65 (1.14‐2.72)	0.02	NM	NM

### 2.6 Statistical analysis

The Forest plot analysis was used to assess the association between CCND2/3 and various cancers. A vertical invalid line (the horizontal coordinate scale is one) was considered as a graphic center. A prism is used to describe the effects and confidence intervals of multiple research. In this study, when the prism intersects the invalid line, suggesting CCND2/3 has no significant prediction for cancer prognosis. When the prism falls to the right of the invalid line, suggesting CCND2/3 predicts high risk of cancers. When the prism falls to the left of the invalid line, suggesting CCND2/3 predicts low risk of cancers. Higgins I^2^ statistic was utilized to quantify the effect of heterogeneity in each eligible study. If the heterogeneity is acceptable (I^2^ < 50% suggested no obvious heterogeneity), the fixed effect model will be adopted; otherwise, a random‐effects model was used instead. In addition, subgroup analysis was adopted to reduce the effects of heterogeneity. Begg's funnel plots and Egger's linear regression test were used to detect the publication bias between all included studies. Sensitive analysis was tested to determine the stability and reliability of the results in this meta‐analysis. All *P*‐values were calculated using a two‐sided test, and *P* < 0.05 was considered statistically significant. STATA 12.0 software (State Corporation, College Station, TX) was utilized to dispose all above statistical analyses.

## 3 RESULTS

### 3.1 The prognostic role and expression of CCND2/3 from available database

**Figure 1:**
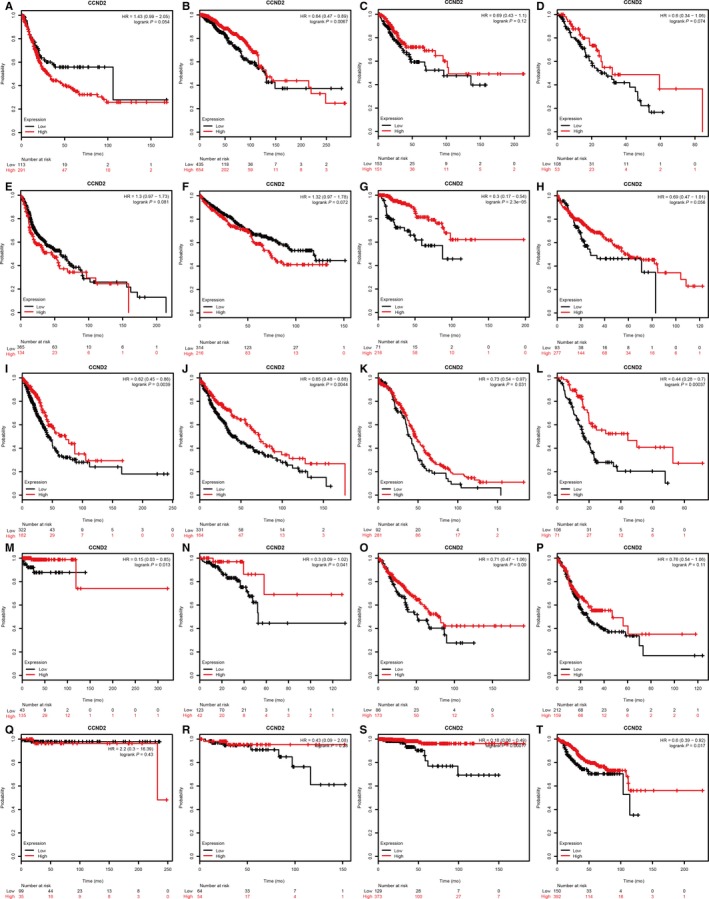
The prognostic role of CCND2 in bladder cancer (A), breast cancer (B), cervical squamous cell cancer (C), esophageal cancer (D), head‐neck squamous cell cancer (E), kidney renal clear cell cancer (F), kidney renal papillary cell cancer (G), liver hepatocellular cancer (H), lung cancer (I), lung squamous cell cancer (J), ovarian cancer (K), pancreatic ductal adenocarcinoma (L), pheochromocytoma and paraganglioma (M), rectum adenocarcinoma (N), sarcoma (O), stomach adenocarcinoma (P), testicular germ cell tumor (Q), thymoma (R), thyroid carcinoma (S), uterine corpus endometrial carcinoma (T)

**Figure 2:**
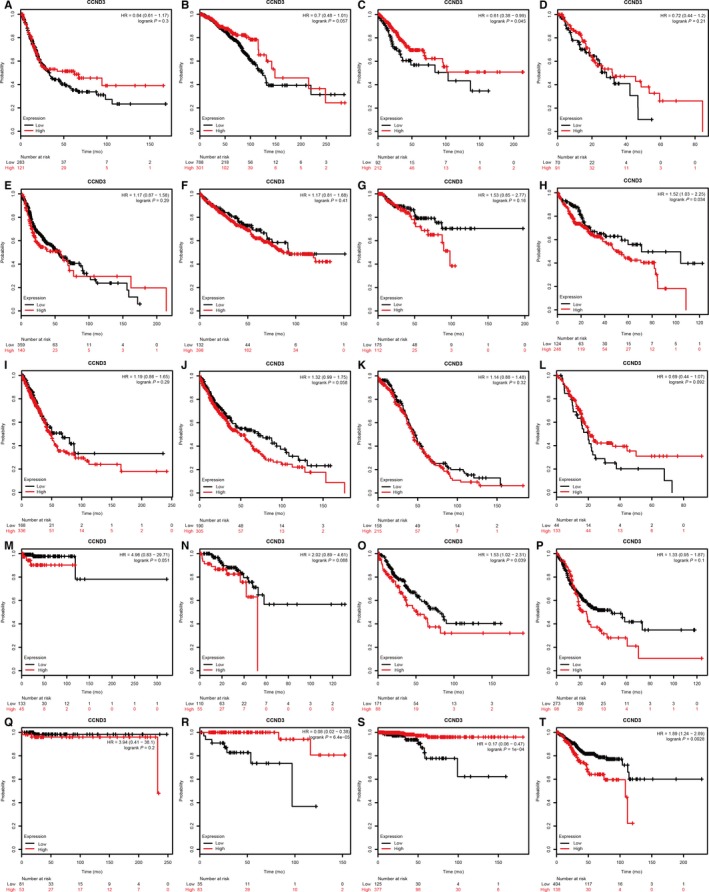
The prognostic role of CCND3 in bladder cancer (A), breast cancer (B), cervical squamous cell cancer (C), esophageal cancer (D), head‐neck squamous cell cancer (E), kidney renal clear cell cancer (F), kidney renal papillary cell cancer (G), liver hepatocellular cancer (H), lung cancer (I), lung squamous cell cancer (J), ovarian cancer (K), pancreatic ductal adenocarcinoma (L), pheochromocytoma and paraganglioma (M), rectum adenocarcinoma (N), sarcoma (O), stomach adenocarcinoma (P), testicular germ cell tumor (Q), thymoma (R), thyroid carcinoma (S), uterine corpus endometrial carcinoma (T)

As shown in Figure 1, CCND2 could be a prognostic biomarker in breast cancer, kidney renal papillary cell cancer, lung cancer, ovarian cancer, pancreatic ductal adenocarcinoma, pheochromocytoma and paraganglioma, rectum adenocarcinoma, thyroid carcinoma, and uterine corpus endometrial carcinoma (*P* < 0.05). CCND3 might be associated with the prognosis in patients with cervical squamous cell cancer or liver hepatocellular cancer or sarcoma or thymoma or thyroid carcinoma or uterine corpus endometrial carcinoma (Figure 2A‐T) (*P* < 0.05). Discrepant mRNA levels of CCND2/3 were observed in normal or tumor tissue in various cancers (Figure S1A,B). In addition, the protein levels of CCND2/3 in various cancers included in this meta‐analysis were also discrepant (Figure S2A‐D). Due to the fact that contradictory results of prognosis value or expression of CCND2/3 exist in various tumors, a meta‐analysis for CCND2/3 prognostic role in multiple human malignant neoplasms need to be conducted to clarify the real association.

### 3.2 Studies characteristics

**Figure 3:**
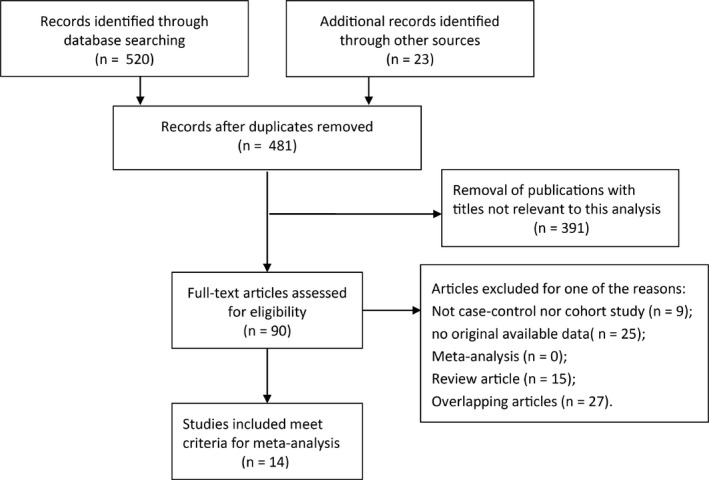
The flowchart of literature search and selection procedure

The search result yielded 543 studies from PubMed, EMBASE, and Web of Science. Based on the inclusion and exclusion criteria, a total of 14 studies were selected for our meta‐analysis for a further evaluation.^6^, ^11^, ^12^, ^17^, ^25^, ^26^, ^27^, ^28^, ^29^, ^30^, ^31^, ^32^, ^33^, ^34^ The flow chart of the literature search and screening process were shown in Figure 3. All of the baseline characteristics of the studies related to the prognosis of human malignant neoplasms were listed in Tables 1 and 2. Among the selected studies, 11 studies reported patient OS, 9 studies focus on DFS/RFS/PFS, and 5 studies investigated OS as well as DFS or PFS. Six studies were conducted in Asians, 8 studies focused on Caucasians, and two studies contained mixed population. In addition, malignant neoplasms included in this meta‐analysis are gastric cancer, NSCLC, colorectal cancer, breast cancer, ovarian cancer, and bladder cancer. The source of HR and 95% CI was extracted from survival curves or article reports.

### 3.3 OS associated with CCND2 expression

**Figure 4:**
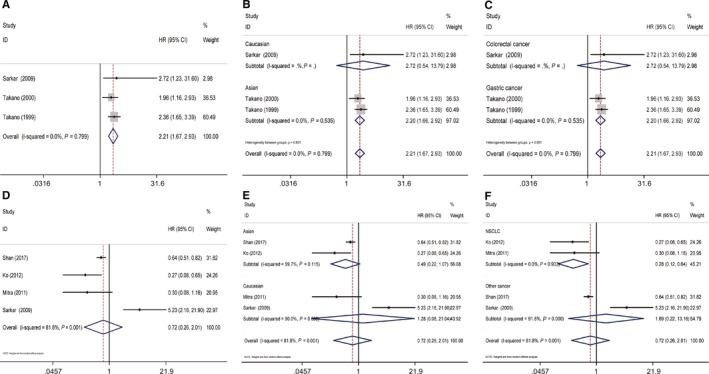
Forest plots of OS or DFS/RFS/PFS in association with CCND2 in various cancers. (A) The overall group, OS; (B) the dominant ethnicity subgroup, OS; (C) The tumor subgroup, OS; (D) The overall group, DFS/RFS/PFS; (E) the dominant ethnicity subgroup, DFS/RFS/PFS; (F) The tumor subgroup, DFS/RFS/PFS

We have included 3 studies in the analysis for the association between OS and CCND2 expression. Sensitivity analysis indicated if the data toward OS from Shan et al^18^ was included in our review, they might have significant impact on the pooled significance. After deleted the data, the results indicated CCND2‐positive expression was an important adverse predictor of OS in cancer patients (pooled HR = 2.21, 95% CI: 1.67‐2.93, *P* = 0.799) (Figure 4 **A**). In ethnicity subgroup, high expression of CCND2 was related to unfavorable OS in Asians (HR = 2.20, 95% CI: 1.66‐2.92, *P* = 0.535) (Figure 4B). When stratified by tumor type, increased CCND2 expression correlated with lower OS in gastric cancer (HR = 2.20, 95% CI: 1.66‐2.92, *P* = 0.535) (Figure 4C). Due to limited numbers of included studies, correlation between CCND2 and OS in other ethnicities or tumor types have not been further analyzed.

### 3.4 DFS/RFS/PFS associated with CCND2 expression

There were 4 out of 14 studies that were adopted to analyze the association between CCND2 expression and DFS/RFS/PFS. CCND2 expression was not a significant predictor of DFS/RFS/PFS according to the quantitative synthesis results (HR = 0.72, 95% CI: 0.26‐2.01, *P* = 0.001) (Figure 4D). In ethnicity subgroup, no significant results were observed in either Asians or Caucasians (Figure 4E). In tumor subgroup, high expression of CCND2 predicted low risk of cancer progression (HR = 0.28, 95% CI: 0.12‐0.64, *P* = 0.903) in NSCLC patients, while no significant association was found in other tumor types (HR = 1.69, 95% CI: 0.22‐13.16, *P* < 0.001) (Figure 4F).

### 3.5 OS associated with CCND3 expression

**Figure 5:**
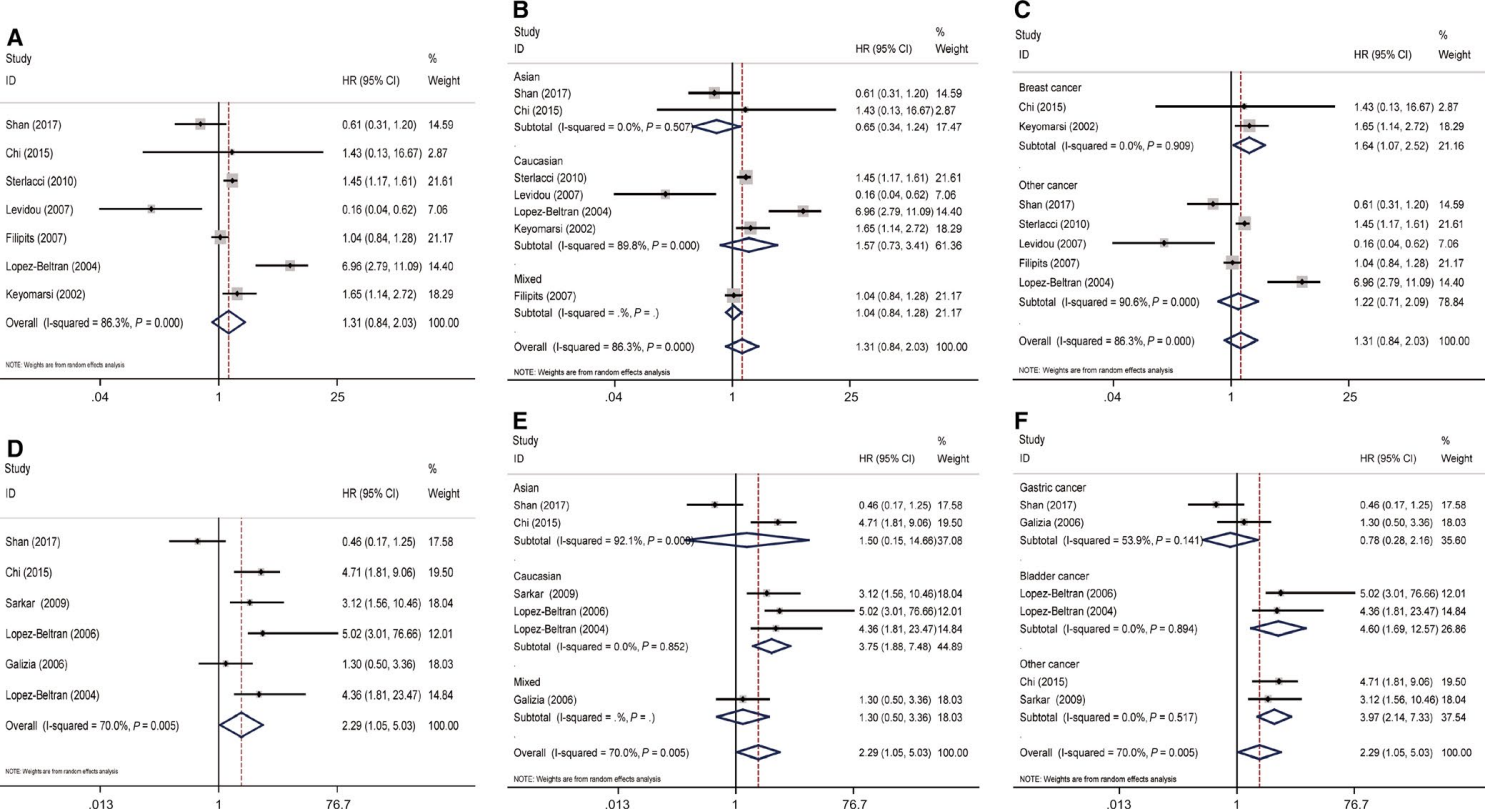
Forest plots of OS or DFS/RFS/PFS in association with CCND3 in various cancers. (A) The overall group, OS; (B) the dominant ethnicity subgroup, OS; (C) The tumor subgroup, OS; (D) The overall group, DFS/RFS/PFS; (E) the dominant ethnicity subgroup, DFS/RFS/PFS; (F) The tumor subgroup, DFS/RFS/PFS

In the 7 studies that were included to analyze the relationship between OS and CCND3 expression, the pooled HR of these studies was 1.31 (95% CI: 0.84‐2.03, *P* < 0.001), which indicated there was no significant association between OS and CCND3 expression. (Figure 5A) Ethnic subgroup analysis for OS also revealed CCND3 failed to predict OS level in Asians, Caucasians, or mixed population (Figure 5B). In tumor subgroup, CCND3 predicted lower OS in breast cancer (HR = 1.64, 95% CI: 1.07‐2.52, *P* = 0.909), while no significant association was found in other cancers (HR = 1.22, 95% CI: 0.71‐2.09, *P* < 0.001) (Figure 5C).

### 3.6 DFS/RFS/PFS associated with CCND3 expression

A total of 6 studies analyzed DFS/RFS/PFS. The results indicated CCND3 overexpression predicted higher risk of cancer progression (HR = 2.29, 95% CI: 1.05‐5.03, *P* = 0.005) (Figure 5D). In ethnicity subgroup, the promoted cancer progression role of CCND3 was observed in Caucasians (HR = 3.75, 95% CI: 1.88‐7.48, *P* = 0.852) (Figure 5E). In tumor subgroup, CCND3 revealed oncogenic role in bladder cancer (HR = 4.60, 95% CI: 1.89‐12.57, *P* = 0.894) (Figure 5F).

### 3.7 Sensitivity analyses

**Figure 6:**
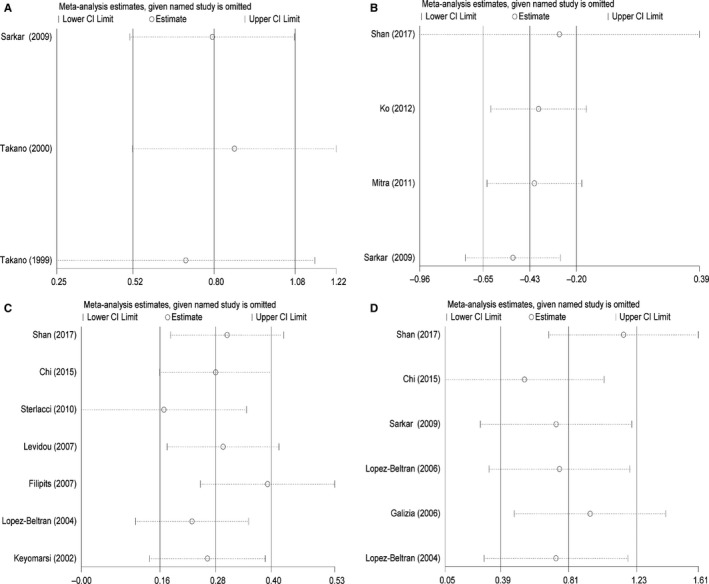
Sensitivity analysis of each included study. (A) OS for individual studies, CCND2; (B) DFS/RFS/PFS for individual studies, CCND2; (C) OS for individual studies, CCND3; (D) DFS/RFS/PFS for individual studies, CCND3

Sensitivity analysis was utilized to determine the robustness and to evaluate the stability of results. The sensitivity analysis for each included study was shown in Figure 6A‐D. The results showed that our results were comparatively credible and stable.

### 3.8 Publication bias

**Figure 7:**
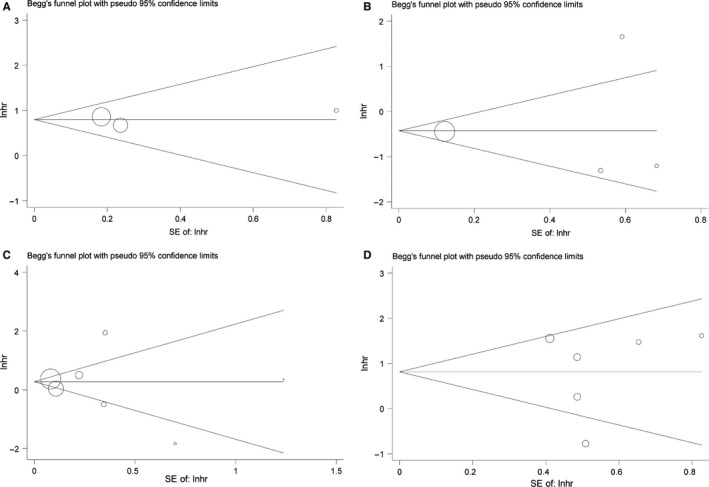
Begg's funnel plots of the publication bias. (A) OS for individual studies, CCND2; (B) DFS/RFS/PFS for individual studies, CCND2; (C) OS for individual studies, CCND3; (D) DFS/RFS/PFS for individual studies, CCND3

Begg's funnel and the Egger's test were applied to assess the publication bias of the literature. The funnel plots of the publication bias are shown in Figure 7A‐D. The *P* values of Begg's test and the *P* values of Egger's test were all greater than 0.05, indicating no publication bias in this study.

## 4 DISCUSSION

Accumulation of evidences has demonstrated aberrant D‑type cyclins expression could cause a number of potentially oncogenic responses and somewhat influence the prognosis of cancer patients.^4^ Activation of cyclin D–cyclin‐dependent kinase 4 (CDK4) or CDK6 contributes to cell cycle progression via the substrate phosphorylation, including a broader range of cell cycle‐related proteins.^1^ In addition, increasing evidence over the past two decades have revealed that except for the well‐known role of promoting cycle entry and progression, D‑type cyclins also possessed additional functions, such as DNA damage repair, gene transcription, cell death, cell differentiation, and metabolism.^35^ CCND2 overexpression is associated with greater depth of cancer invasion, the presence of lymph node metastasis, and poor prognosis in gastric cancer.^25^ Although cyclin D2 is overexpressed in certain cancers, reduced cyclin D2 is also observed in breast, pancreatic, and prostate cancer.^36^, ^37^ These studies indicated CCND2 predicted diverse, even opposing outcome. As for CCND3, several studies also revealed different predictor role in various cancers.^30^, ^33^ The discrepancies between these studies highlight the necessity of evaluating the prognostic significance of CCND2 and CCND3 in various human cancers. Meanwhile, we found discrepant expression or prognostic value of CCND2/3 in various tumors from available databases. Therefore, we conducted this meta‐analysis to explore the prognostic role of CCND2 and CCND3 in multiple human malignant neoplasms.

To the best of our knowledge, this meta‐analysis is the first review to clarify the prognostic role of CCND2/3 in multiple human malignant neoplasms using meta‐analysis. The associations between CCND2/3 and prognostic outcomes (OS, DFS/RFS/PFS) of patients with various cancers were explored systemically. Our results indicated CCND2/3 played an oncogenic role in all cancer patients. We found high expression of CCDN2 was associated with reduced OS in gastric cancer patients, whereas it was related with favorable outcome in NSCLC patients. In addition, CCND3 overexpression was an adverse predictor of OS in breast cancer. Meanwhile, CCND3‐positive expression was correlated with shorter DFS/RFS/PFS in bladder cancer. These results indicated CCND2/3 could be promising biomarkers and novel therapeutic targets for various cancers. When stratified by ethnicity, only Asians revealed significant association between CCDN2 expression and OS level. As for CCND3, significant association between CCND3 expression and DFS/RFS/PFS level was only observed in Caucasians. Thus, CCND2/3 expression in the patients above might be useful for prognosis prediction. Sensitivity analyses and publication bias were performed to make sure that all included studies were robust and stable.

Cyclin D2 is a direct target gene of proto‐oncogene Myc and links growth signaling with the nuclear export of p27, contributing to the progression of the cell cycle through the G(0)‐G(1) transition.^39^ Several studies have suggested hypermethylation of CCND2 promoter induced the deregulated of CCND2 function in gastric cells and primary gastric carcinomas. CCND2 promoter hypermethylation accompanied by the loss of CCND2 expression may suggest an alternative gastric carcinogenesis pathway.^40^ In colon tumors, cyclin D2 was overexpressed in 53% of the case and cyclin D2 overexpression may be related to a higher TNM stage of the tumor, which reveals a potential metastatic role for CCND2.^41^ Our results indicated that CCND2 overexpression was an adverse predictor in colorectal cancer and gastric cancer. Local or distant recurrences of breast cancer is a tricky problem in patients after adopting adjuvant therapy. The reason for recurrences of breast cancer is that a small number of tumor cells resist the effects of adjuvant therapy and then proliferate in local or distant region, and this phenomenon exhibits cyclin D1‐CDK4 dependent proliferation.^42^ Previous studies showed that Cyclin D1 and D3 are overexpressed in primary invasive breast cancers and human breast cancer cell lines.^43^ Some studies found CCND1 downregulation have no effect on cancer cell proliferation, which confused many researchers. Zhang et al^43^ reported the lack of CCND1 was associated with a compensatory upregulation of CCND3 and the inhibition of both CCND1 and CCND3 could be a suitable strategy for breast cancer prevention and therapy. CCND3 expression were positively associated with ER, PR, and negatively correlated with tumor differentiation status. Univariate and multivariate analyses were all revealed CCND3 expression was associated with higher risk of recurrence, which suggested Cyclin D3 might be an independent prognostic factor for breast cancer patients.^11^ In our meta‐analysis, positive‐CCND3 is associated with reduced OS in breast cancer patients. CCND3 play an oncogenic role in breast cancer, which is consistent with the viewpoints above.

There are many strengths to the study. Sensitivity and publication bias analysis were conducted to ensure robustness of the study. In addition, this is the first systematic review to address the prognostic role of CCND2/3 in multiple human malignant neoplasms. To a certain extent, limitations should also be addressed. First, it was difficult to establish a standard expression cutoff point due to varied cutoff points in the selected studies. This could lead to bias in the effectiveness of CCND2/3 as a prognostic factor in cancer patients. Second, the retrospective nature of the majority of the studies could hinder a clear impact on group baseline features. Third, the number of included studies in the stratified analyses was not enough, leading to the limited statistical power to explore the real relationship. Last but not least, cancer is a kind of multifactorial disease, related to complex interactions between genetic and environment factors. It is so complicated that the investigation of gene expression cannot predict the prognosis of cancers accurately. Considering these limitations, our results should be interpreted rigorously, and more attention should be paid to the function of CCND2/3 in various carcinomas in large multicentric studies.

## 5 CONCLUSIONS

In summary, this meta‐analysis suggested that CCND2 played an oncogenic role in gastric cancer, whereas it could also be a tumor suppressor in NSCLC. In addition, CCND3 was an adverse prognostic factor in breast cancer and bladder cancer. These results indicated CCND2/3 could be promising biomarkers and novel therapeutic targets for patients with malignant neoplasms, and the biological functions of CCND2/3 are of great research value of the subject.

## CONFLICT OF INTEREST

The authors declare that they have no competing interests.
