# Supplementary figures and images for: Prognostic role of cyclin D2/D3 in multiple human malignant neoplasms: A systematic review and meta‐analysis
Source: Cancer Med. 2019 Apr 5;8(6):2717–29. doi: 10.1002/cam4.2152 (PMC6558476; doi:10.1002/cam4.2152)

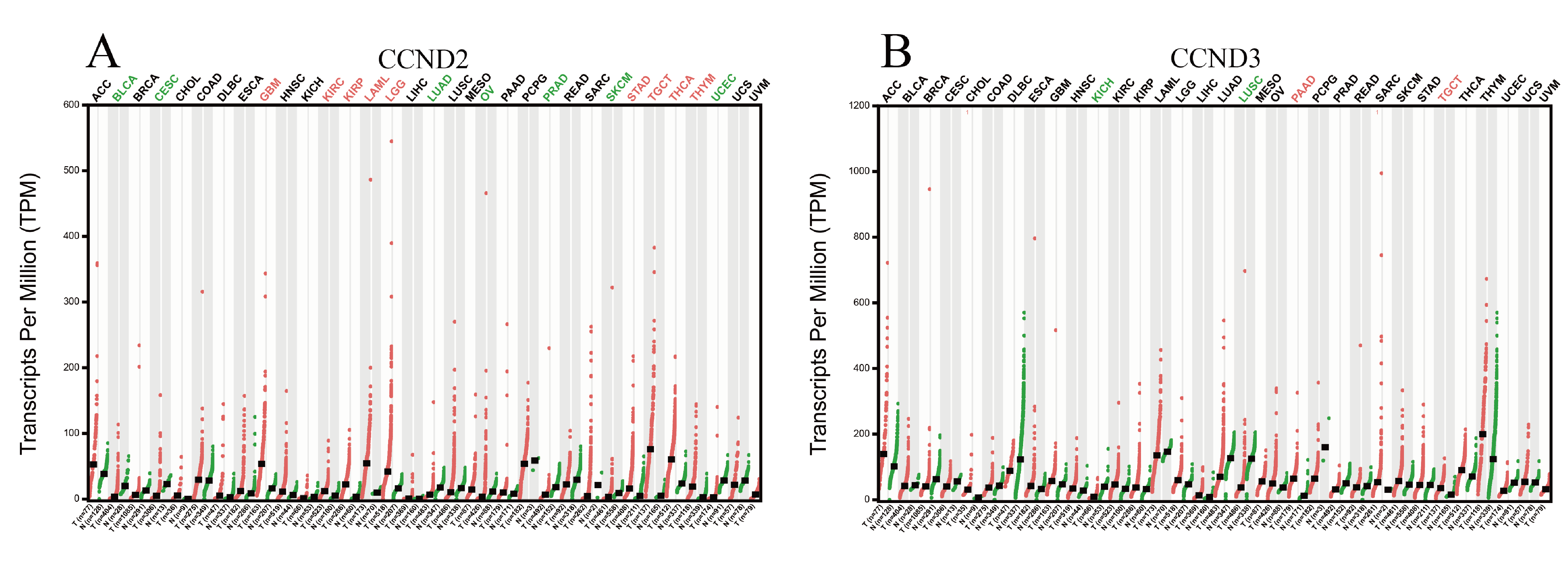

Supplement: Supplementary file 1 [file CAM4-8-2717-s001.tif]

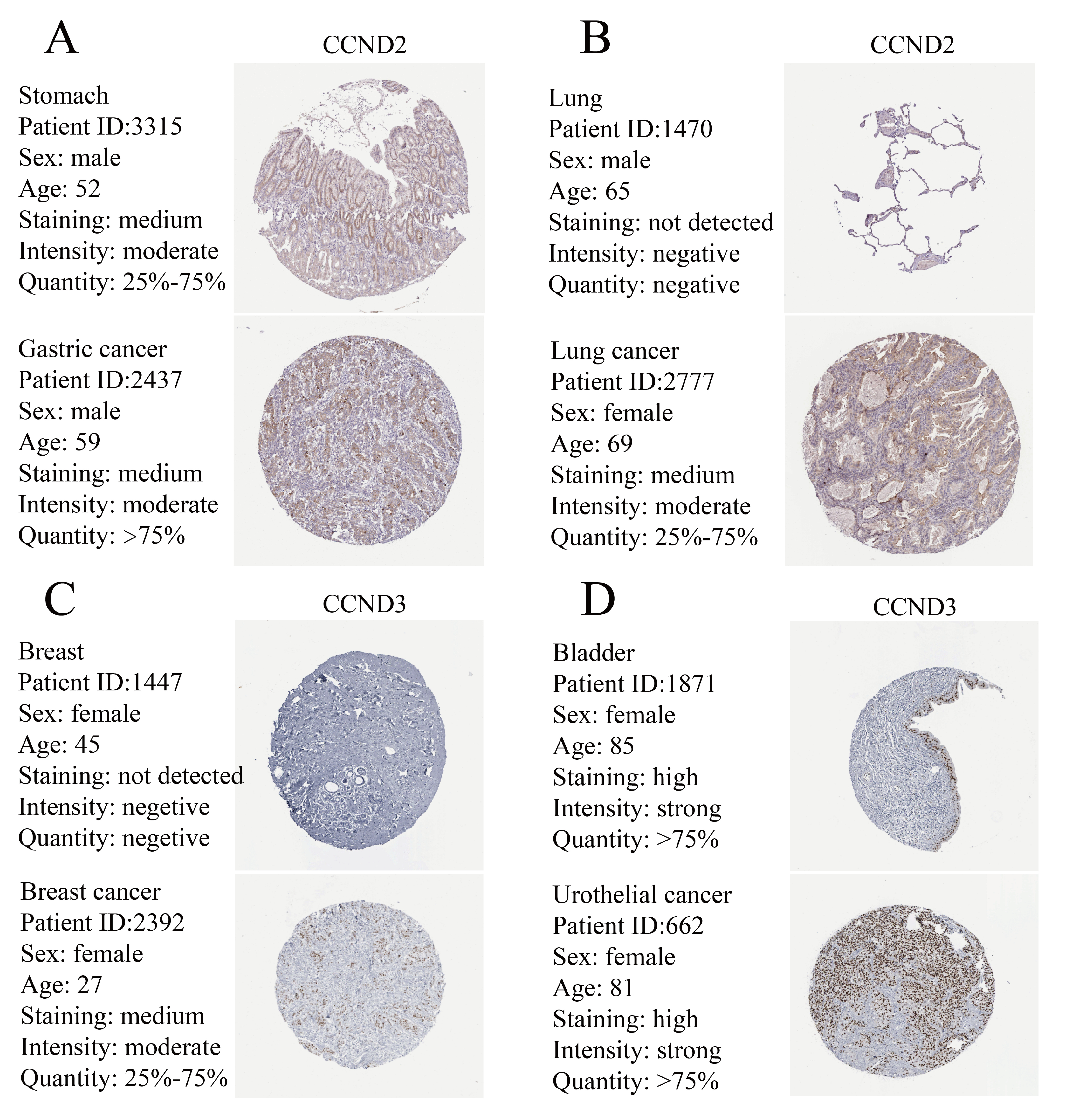

Supplement: Supplementary file 2 [file CAM4-8-2717-s002.tif]
